# Supplementary figures and images for: Sympathetic innervation of interscapular brown adipose tissue is not a predominant mediator of oxytocin-elicited reductions of body weight and adiposity in male diet-induced obese mice
Source: Front Endocrinol (Lausanne). 2024 Jul 31;15:1440070. doi: 10.3389/fendo.2024.1440070 (PMC11321955; doi:10.3389/fendo.2024.1440070)

## SHAM

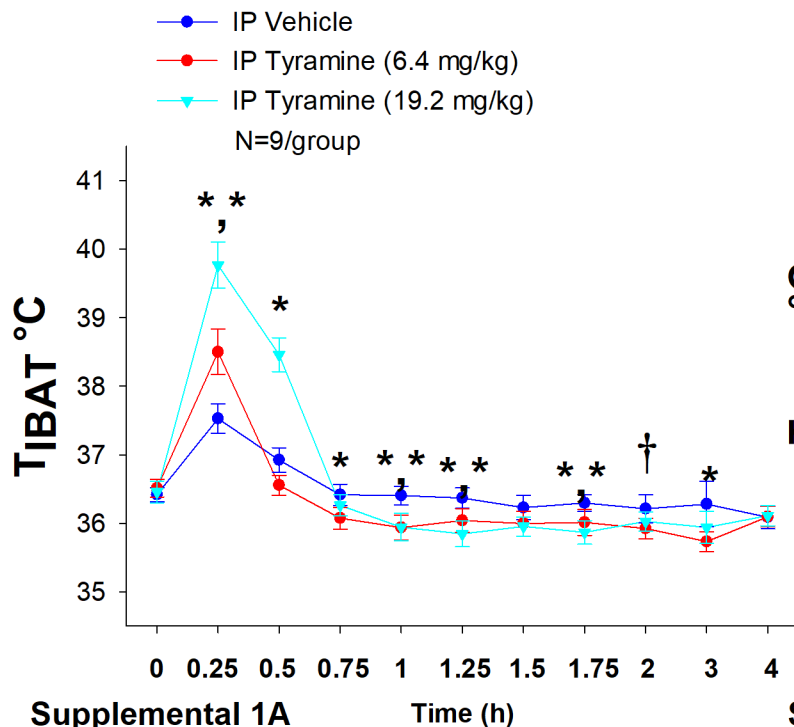

## DENERVATED

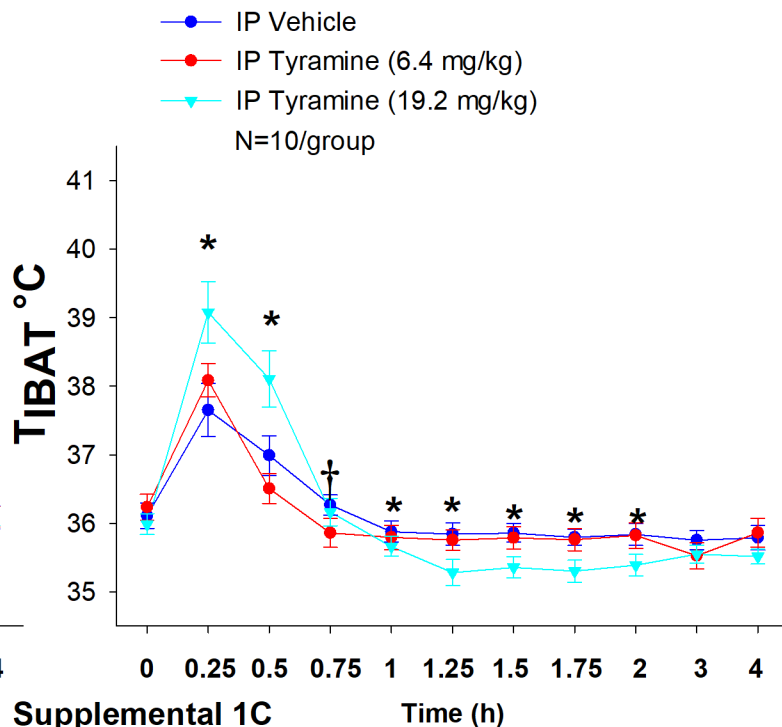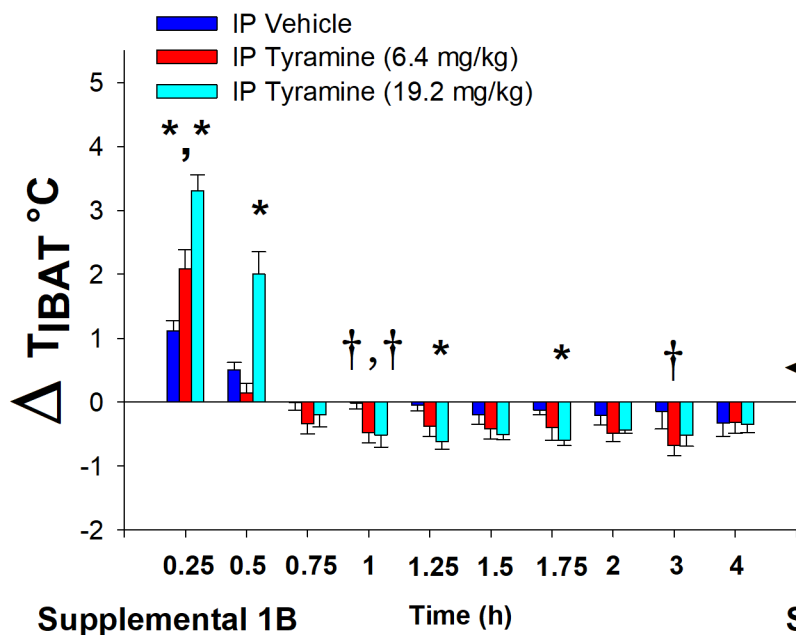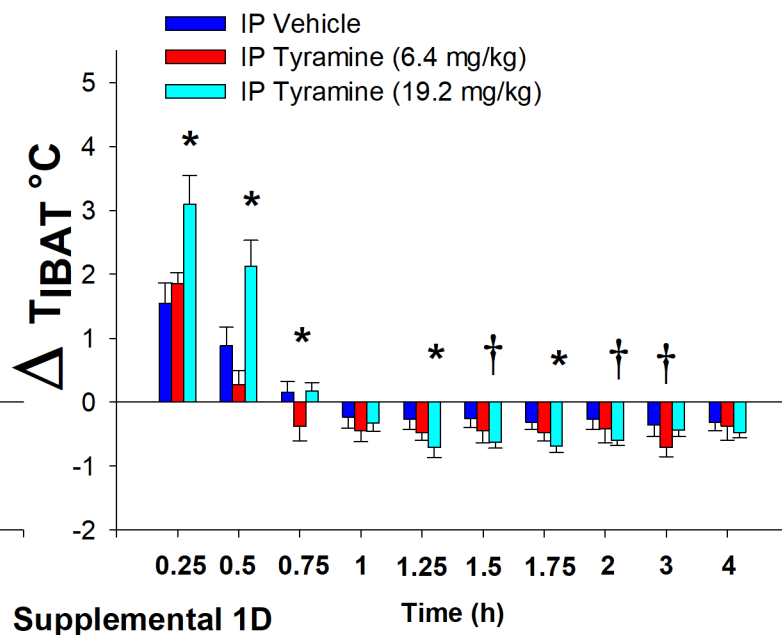

Supplement: Supplemental Study 1 — Determine if surgical denervation of IBAT changes the ability of IP tyramine to increase TIBAT in DIO mice. The goal of this study was to determine if IP tyramine-elicited increase in TIBAT requires intact SNS outflow to IBAT in DIO mice. We selected doses of tyramine based on previous studies (105). By design, mice were DIO as determined by both body weight (49.5 ± 1.1 g) and adiposity (13.9 ± 0.8 g fat mass; 31.2 ± 1.4% adiposity) after maintenance on the HFD (60% kcal from fat; N=9-10/group) for approximately 4.25 months prior to sham/denervation procedures and implantation of temperature transponders underneath IBAT. Mice from Study 4 were used in this study and were otherwise treated identically to those used in Study 4. Supplemental Study 1. In sham mice, tyramine (6.4 mg/kg) increased TIBAT at 0.25-h post-injection while the higher dose (19.2 mg/kg) increased TIBAT at 0.25 and 0.5-h post-injection (P<0.05; Supplementary Figure 1A ). The lower dose also reduced TIBAT at 0.75, 1, 1.25, 1.75, and 3-h post-injection and tended to reduce TIBAT at 2-h post-injection. The higher dose reduced TIBAT at 1, 1.25 and 1.75-h post-injection. Similar findings were apparent when measuring change in TIBAT relative to baseline TIBAT ( Supplementary Figure 1B ). In denervated mice, tyramine (6.4 mg/kg) was not effective at increasing TIBAT but tended to reduce TIBAT at 0.75-h post-injection (P<0.05; Supplementary Figure 1C ). The higher dose (19.2 mg/kg) stimulated TIBAT at 0.25 and 0.5-h post-injection and reduced TIBAT at 1.25, 1.5, 1.75 and 2-h post-injection (P<0.05). Similar findings were also apparent when measuring change in TIBAT relative to baseline TIBAT ( Supplementary Figure 1D ). [file DataSheet_1.pdf]

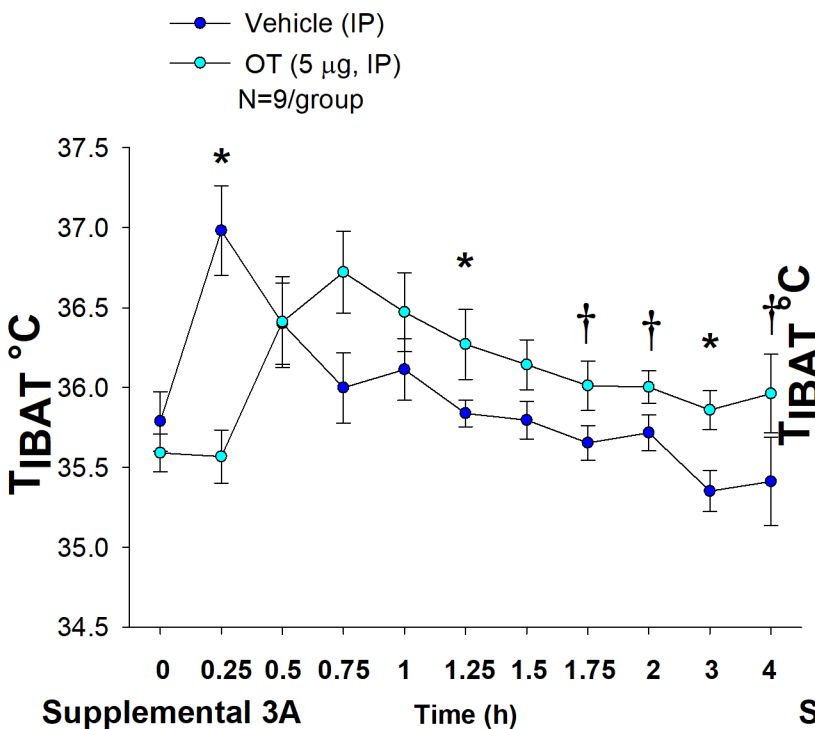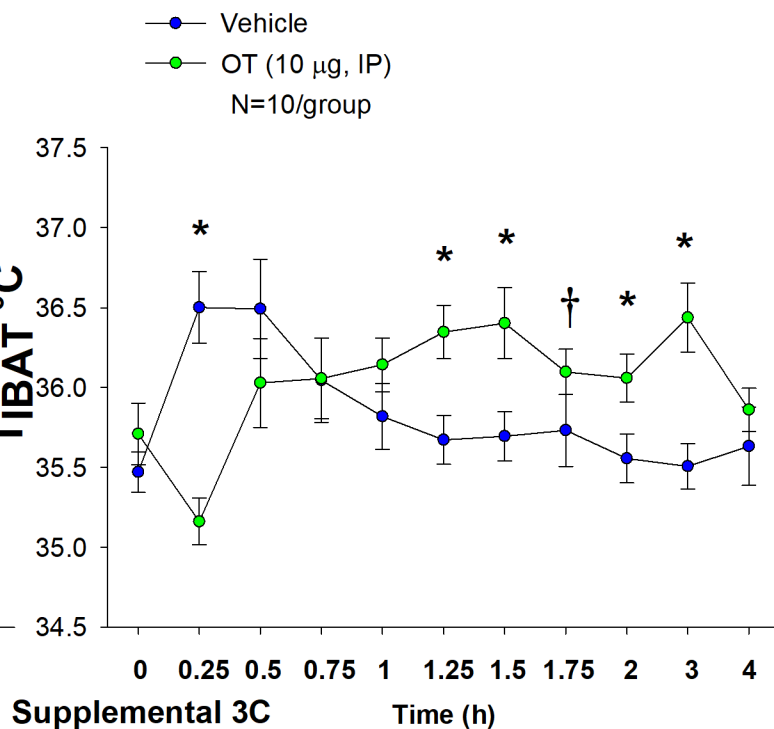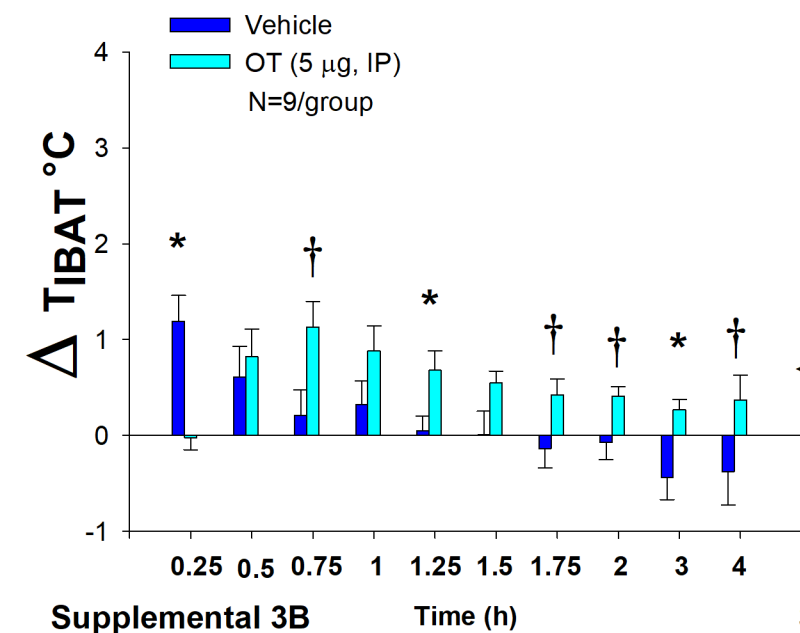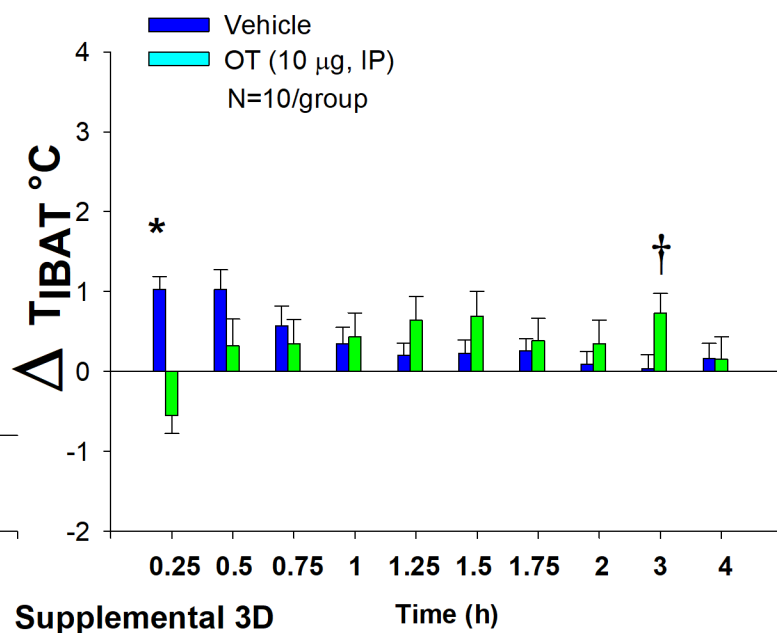

Supplement: Supplemental Study 3 — Determine if a centrally (4V) effective dose of OT can increase TIBAT when given into the periphery of lean mice. The goal of this study was to determine if systemic administration of OT (IP) can increase TIBAT at a dose that was effective when given into the 4V in lean mice. By design, mice were lean (31.2 ± 0.8 g) at study onset after maintenance on the chow (16% kcal from fat; N=10/group) for approximately 5 months. Mice did not undergo sham or SNS IBAT denervation procedures but were implanted with temperature transponders underneath IBAT and were otherwise treated similarly to those used in Study 4.Supplemental Study 3. In contrast to the more immediate elevation of TIBAT in response to 4V OT (5 μg/μL), IP OT (5 μg/0.200 mL) resulted in a reduction of TIBAT at 0.25-h post-injection followed by increases in TIBAT at 1.25 and 3-h post-injection (P<0.05; Supplementary Figure 3A ). IP OT (5 μg/0.200 mL) also tended to stimulate TIBAT at 1.25, 2 and 4-h post-injection. In addition, we found similar findings were apparent when measuring change in TIBAT relative to baseline TIBAT ( Supplementary Figure 3B ). In addition, a higher dose of OT (10 μg/0.200 mL) produced a similar reduction of TIBAT at 15-min post-injection followed by increases in TIBAT at 1.25, 1.5, 2, and 3-h post-injection (P<0.05; Supplementary Figure 3A ). IP OT (10 μg/0.200 mL) also tended to stimulate TIBAT at 105 min-post-injection. In addition, we found similar findings were apparent when measuring change in TIBAT relative to baseline TIBAT ( Supplementary Figure 3B ). [file DataSheet_3.pdf]
