# Supplementary material for: Sympathetic innervation of interscapular brown adipose tissue is not a predominant mediator of oxytocin-elicited reductions of body weight and adiposity in male diet-induced obese mice
Source: Front Endocrinol (Lausanne). 2024 Jul 31;15:1440070. doi: 10.3389/fendo.2024.1440070 (PMC11321955; doi:10.3389/fendo.2024.1440070)

Effects of Acute 4V Oxytocin on IBAT Temperature in Lean Mice with Intact or Denervated SNS Outflow to IBAT

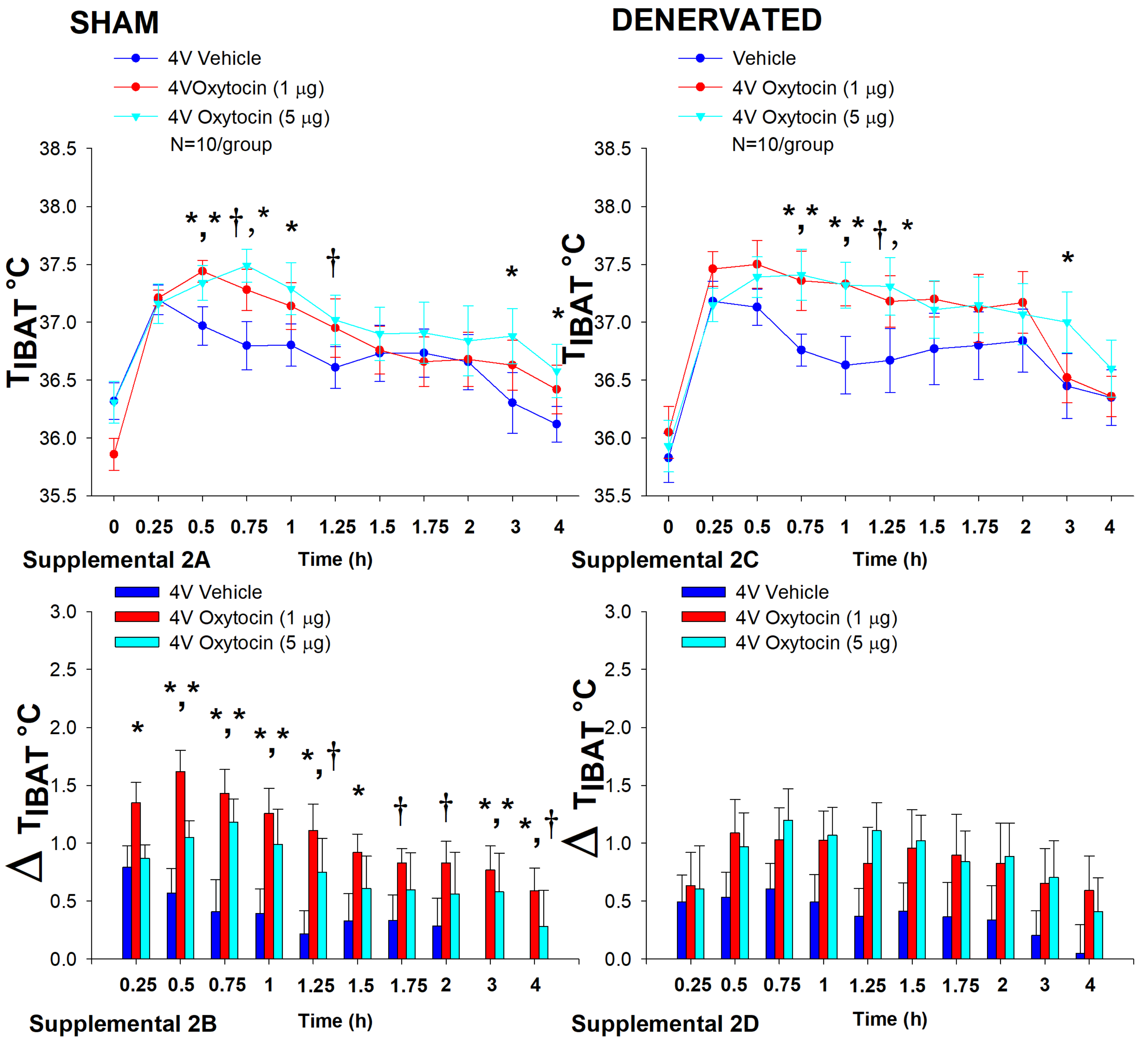

Supplement: Supplemental Study 2 — Determine if surgical denervation of IBAT changes the ability of 4V OT to increase TIBAT in lean mice. The goal of this study was to determine if OT- elicited increase in TIBAT requires intact SNS outflow to IBAT in lean mice. By design, mice were lean as determined by both body weight (29.5 ± 0.7 g) and adiposity (3.3 ± 0.3 g fat mass; 10.4 ± 0.8% adiposity) after maintenance on the chow (16% kcal from fat; N=10/group) for approximately 4-4.25 months prior to sham/denervation procedures and implantation of temperature transponders underneath IBAT. Mice were otherwise treated identically to those used in Study 4. Supplemental Study 2. Only a subset of samples from Study 1B were able to be screened for NE content but all mice were otherwise included in the analysis. In sham mice, 4V OT (5 μg/μL) increased TIBAT at 0.5, 0.75, 1 and 3-h post-injection (P<0.05; Suplemental Figure 2A ) and tended to stimulate TIBAT at 0.75 (1 μg/μL) and 1.25 (5 μg/μL) h-post-injection. In addition, we found similar findings were apparent when measuring change in TIBAT relative to baseline TIBAT ( Supplementary Figure 2B ). Similarly, in denervated mice, 4V OT (5 μg/μL) increased TIBAT at 0.75, 1, 1.25 and 3-h post-injection (P<0.05; Supplementary Figure 2C ) and tended to stimulate TIBAT at 1.25 (1 μg/μL) -h post-injection. In contrast, 4V OT was unable to stimulate a change in TIBAT relative to baseline TIBAT ( Supplementary Figure 2D ). [file DataSheet_2.pdf]
